# Behavioral verification and risk factors of HIV cross-population transmission in China: analysis of national surveillance data 1989–2022

**DOI:** 10.1186/s12879-023-08956-9

**Published:** 2024-01-04

**Authors:** Chang Cai, Houlin Tang, Qianqian Qin, Yichen Jin, Fan Lyu

**Affiliations:** 1 Division of Epidemiology, National Center for AIDS/STD Control and Prevention, Chinese Center for Disease Control and Prevention, Beijing, 102206 China; 2 National Key Laboratory of Intelligent Tracking and Forecasting for Infectious Diseases, National Center for AIDS/STD Control and Prevention, Chinese Center for Disease Control and Prevention, Beijing, 102206 China

**Keywords:** HIV/AIDS, Men who have sex with men, Former plasma donors, People who inject drugs, Heterosexual behavior, Risky behavior, China

## Abstract

**Introduction:**

The dynamic HIV/AIDS epidemic significantly impacts China, particularly affecting injection drug users (IDUs), former plasma donors (FPDs), men who have sex with men (MSM), and those engaging in high-risk heterosexual behavior (HRHB). This study specifically focuses on identifying the risk factors and influences that drive the spread of HIV among these population groups by performing a comprehensive analysis of contact histories of individuals diagnosed with HIV.

**Methods:**

Data for this research were gathered from China’s HIV/AIDS Comprehensive Response Information Management System (CRIMS). Contact histories were described using bar and venn diagram. Trend in engaging in HBRB among MSM were identify potential change using the Cochran-Armitage test. Logistic regression was employed to analyze the factors influencing HBRB in MSM.

**Results:**

From 1989 through to 2022, a total of 1,457,218 individuals aged 15 years or older in China, who reported being infected with HIV, indicated they had one or more types of contact histories including injecting drug use, male homosexual behavior, commercial plasma donation, and high-risk heterosexual behavior. Among these, 97.0% reported a single type of contact history, while 3.0% reported having multiple contact histories. Of those with multiple contact histories, 98.0% (42,258 individuals) had engaged in HRHB. Among all HIV-infected IDUs, MSM, and FPDs, their respective proportions of engagement in HRHB were 11.8%, 5.7% and 6.2%. Prior to 2012, most were reported to be IDUs; however, subsequent to this, most reported being MSM. Factors that heightened the risk of engaging in HRHB among HIV-infected MSM included being of age between 25–34 years [adjusted odds ratio (*AOR*) = 1.29] or 35–44 years (*AOR* = 1.22), marital status such as being married (*AOR* = 1.23) or being divorced/widowed (*AOR* = 1.17), belonging to an ethnic minority (*AOR* = 1.29), receiving diagnosis in hospitals (*AOR* = 1.81), residing in rural areas (*AOR* = 1.12), among others. However, the risk of HRHB decreased when age ≥ 55 years (55–64 years: *AOR* = 0.82; ≥ 65 years: *AOR* = 0.64).

**Conclusion:**

The potential for HIV transmission among diverse populations is substantial. As such, it is imperative that strategies are implemented to mitigate the propagation of HIV to the general populace via heterosexual intercourse.

## Background

AIDS has been pervasive in China for over three decades, with the primary route of infection shifting from blood transmission to sexual transmission [1]. Since the first domestic reported cases, a series of national response efforts have been made to reduce the transmission. By the end of 2020, 92.9% of all the alive people living with HIV(PLWH) were receiving antiretroviral therapy(ART), 96.1% of those on ART were able to achieve viral suppression [2]. The transmission risk should be very low under such high coverage and effective ART [3]. However, there were still more than 100 thousand newly diagnosed infections every year, of which, the heterosexual transmission has represented over 70% for the past five years consecutively [1]. A modeling study found that persons who are HIV infected but undiagnosed are most likely to transmit HIV [4], in other words, the risky behaviors before diagnosis should account for most of the transmission. This finding might explain our puzzle well, especially when we consider the persistent problem of late diagnosis in China [5, 6].

What’s more, this theory is particularly right when we used it to explain the shifting of transmission route in the early stage of HIV epidemic in China. As we already known, the HIV infection initially confined to injecting drug users (IDUs) and former plasma donors (FPDs), but now almost exclusively transmitted via sexual contact [7, 8]. Viral gene sequence analysis also supports the notion that HIV has transitioned from IDUs and FPDs to the general population, via heterosexual sex [9]. However, neither the shift of transmission routes nor viral gene distribution can prove direct transmission relationships between different populations. Transmission from one population to another only occurs when an infected individual partakes in two or more distinct high-risk behaviors [10]. And the probability of transmission would be particular large when the multiple high-risk behaviors occurred before ART or before diagnosis.

These behaviors are documented as contact histories when cases are diagnosed and reported within China’s HIV/AIDS Comprehensive Response Information Management System (CRIMS). This study assesses four prominent high-risk behaviors that have significantly impacted HIV's epidemic trend in China [1], and aims to ascertain potential transmission linkages between different populations. This could, in turn, contribute to the enhancement of intervention strategies.

## Methods

### Data collection

China established the HIV/AIDS case reporting system in 1985, when the first foreign case was diagnosed. It was a nationwide real-time reporting system, and developed into web-based Comprehensive Response Information Management System (CRIMS) in 2005 [11]. China’s laws on the prevention and treatment of infectious diseases designates AIDS as a class B notifiable infectious disease. Therefore, all newly identified cases of HIV infection are required to be reported through this system by trained individuals from all medical units at the county, city, and provincial levels. Demographic information and data on high-risk behaviors were collected using standardized case report forms. Staffs from local CDCs and National Center for AIDS/STD Control and Prevention (NCAIDS) double evaluate and identify mistakes in logic and duplication to ensure that the system can obtain accurate information throughout the country [12, 13].

Study data was extracted from CRIMS and variables incorporated age, education level, marital status, ethnicity, residence, transmission routes, diagnosis locations, the year of diagnosis, and contact histories.

### Participants

We retrospectively enrolled all people included in the CRIMS database diagnosed with HIV from 1989 to 2022 (because the first domestic case was detected in 1989). The inclusion criteria were (a) age ≥ 15 years at diagnosis (small proportion of cases and different way of information collection), (b) reported partaking in one or more of the following behaviors: injection drug, male homosexual sex, commercial plasma donation, and high-risk heterosexual sex. Participants who cannot remember or refused admitting high-risk behaviors were excluded.

Waiver of informed consent was granted because this analysis used existing data collected during the course of routine surveillance under the Infectious Diseases Act in China. This study was approved by the Institutional Review Board of the National Center for AIDS/STD Control and Prevention, Chinese Center for Disease Control and Prevention(X140121318). The data obtained complied with relevant data protection and privacy regulations and individual identifiers were removed.

### Contact history

For HIV-infected individuals, in most cases, the infectious sources are uncertain. Contact histories mean the experiences may expose individuals to HIV. It was collected once the individuals were diagnosed, and serviced as a most important evidence for assessing the transmission routes. Under the variable of contact history in the CRIMS, there are 13 options can be selected single or multiple, including high-risk behaviors and some invasive medical practices, in which, the risk of acquiring HIV is low but cannot be completely ignored.

According to the contact history of a diagnosed case, the interviewers assess the most possible transmission route. And the assessment is simple if the contact history is single, otherwise, the transmission risk for each kind of behaviors should be taken into account [14]. The principles for assessment were noted below the reporting form. What’s more, a certain case gets infected via an exclusive behavior, however, would transmit HIV via different kinds of behaviors.

### Definitions

High-risk heterosexual behaviors (HRHB) encapsulate all unprotected heterosexual activities occurring outside the context of marriage or a consistent sexual relationship. For more specific, it includes commercial heterosexual contact (selling or buying sex) and non-marital non-commercial heterosexual contact (having sex with transient or casual heterosexual partners) [15]. FPDs denote individuals who engaged in illicit plasma selling during the 1990s [16]. We characterize HIV-infected individuals who departed from their county of birth at the time of diagnosis as migrant people. According to the classification by the National Bureau of Statistics [17], the provinces are categorized into east, central, and west regions for the purposes of our study.

### Statistical analysis

Numbers and proportions were used to describe the four kinds of contact histories which we interested in, and they were also depicted by bar and venn diagram. The occurrence of HBRB among different groups were compared by years. Demographic variables were categorized and presented as number and percentage, using the entire diagnosed HIV-infected MSM as the denominator.

Trend in engaging in HBRB among MSM were identify potential change using the Cochran-Armitage test with an assumed α of 0.05. A multivariate binary logistic regression model was utilized to examine factors associated with HRHB among MSM. For all analyses, *P*-values were two-sided. We employed a threshold of *P* < 0.05 to indicate statistical significance.

SPSS (version 24, IBM Inc., Armonk, NY, USA) and Microsoft Excel 2019 (Microsoft Corp 2019) were adopted for statistical analysis.

## Results

### Contact histories and their overlap

**Fig. 1:**
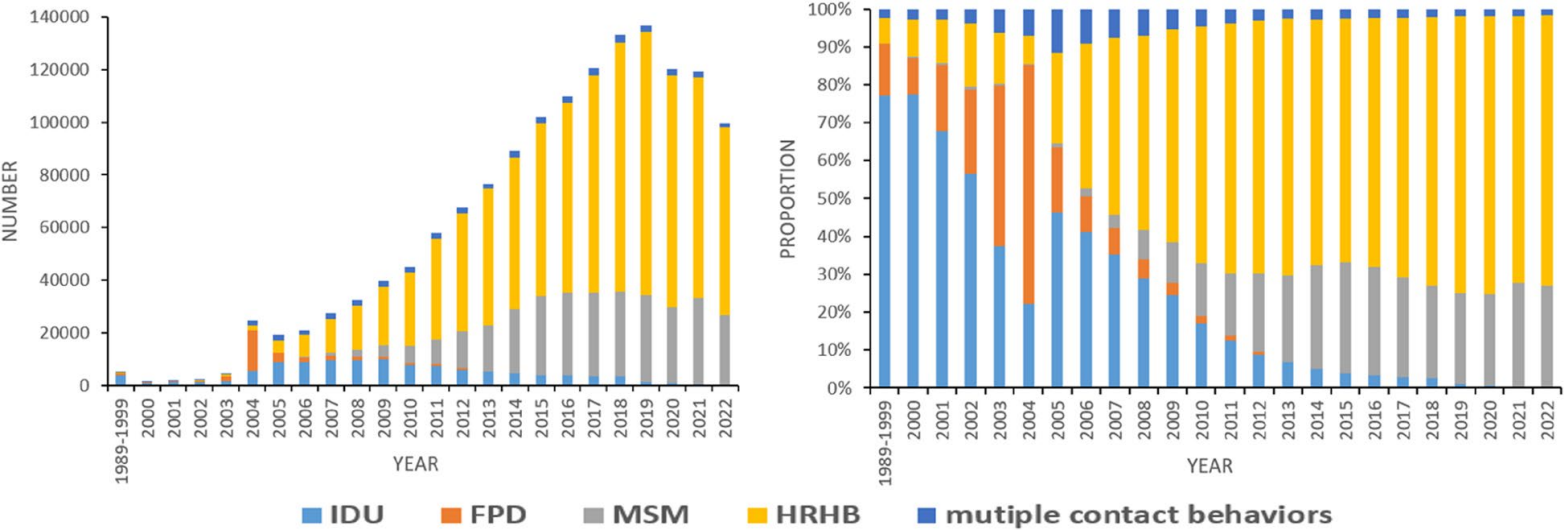
Numbers (left) and proportions (right) of individuals with HIV, categorized by different contact histories across the years 1989–2022 in China. Abbreviations: IDU = injection drug users; FPD = former plasma donors; MSM = men who have sex with men; HRHB = high-risk heterosexual behavior

**Fig. 2:**
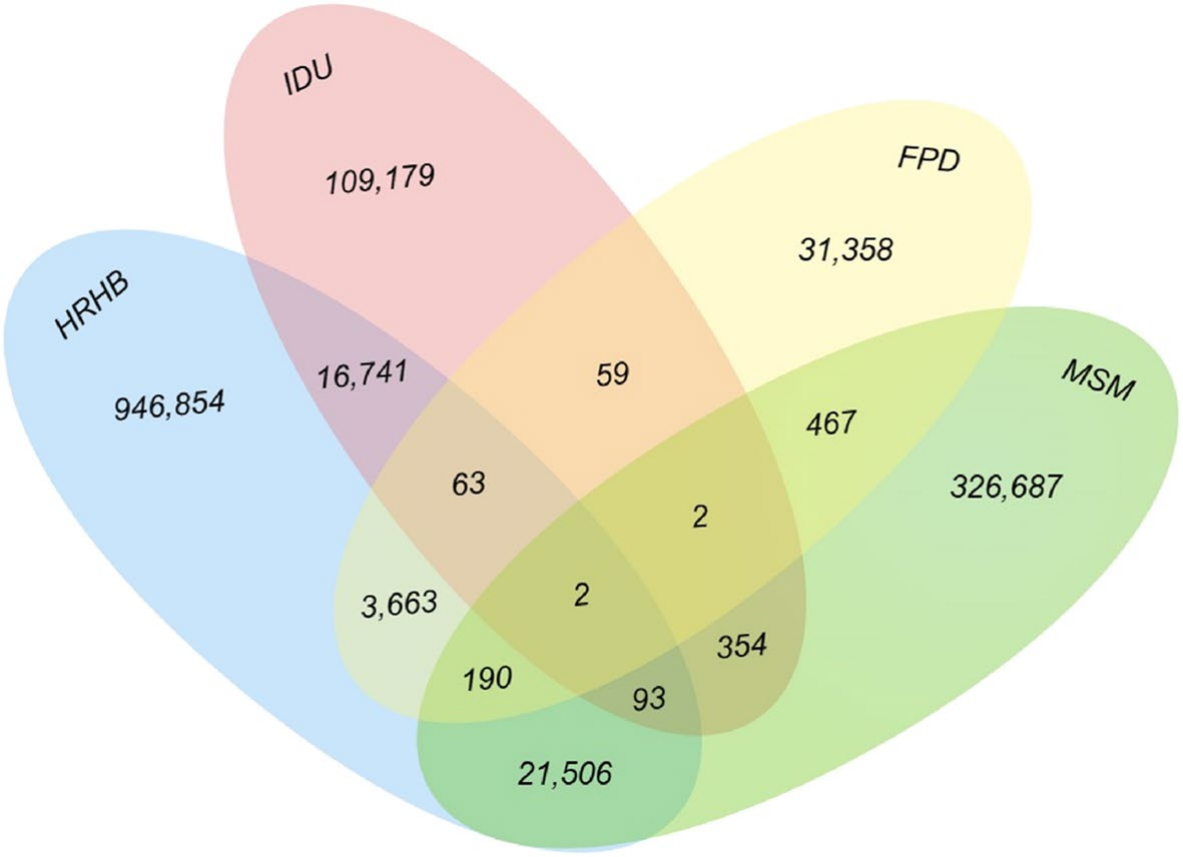
Composition of HIV-infected individuals in China who have engaged in risky behaviors, from 1989 to 2022. Abbreviations: IDU = injection drug users; FPD = former plasma donors; MSM = men who have sex with men; HRHB = high-risk heterosexual behavior

Between 1989 and 2022, 1,457,218 HIV-positive individuals aged 15 years and older in China reported experiencing at least one of the contact histories previously discussed. Of these individuals, 1,414,078 (97.0%) reported single contact history, 42,790 (2.9%) to two contact histories, and 350 (0.02%) to three or more (Figs. 1 and 2). Among the 43,140 respondents who reported multiple contact histories, 21,791 (50.5%) were men who had sexual encounters with other men and engaged in HRHB with women, 16,899 (39.2%) had histories of injecting drugs and HRHB, and 3,918 (9.1%) reported commercially donating plasma as well as HRHB. A minimal overlap was observed among the behaviors of plasma donation, drug injection, and homosexuality (Fig. 2).

Most of the respondents with single or multiple contact histories were male (80.7%), most MSM, IDUs and FPDs were diagnosed before the age of 45, but > 45 at diagnosis were more common among the HRHB group (Table 1).

**Table 1:** Demographics of HIV-infected individuals with different contact history

	Total(%)	Single contact history				Multiple contact histories(%)
		MSM(%)	HRHB(%)	IDU(%)	FPD(%)	
Total	1,457,218	326,687	946,854	109,179	31,358	43,140
Gender						
Male	1,175,706(80.7)	326,687(100.0)	695,030(73.4)	96,193(88.1)	18,668(59.5)	39,128(90.7)
Female	281,512(19.3)	0(0.0)	251,824(26.6)	12,986(11.9)	12,690(40.5)	4,012(9.3)
Age (years)						
15–24	189,580(13.0)	94,139(28.8)	72,608(7.7)	15,182(13.9)	259(0.8)	7,392(17.1)
25–34	370,214(25.4)	118,376(36.2)	174,487(18.4)	52,526(48.1)	6,944(22.1)	17,881(41.4)
35–44	297,933(20.4)	57,679(17.7)	181,479(19.2)	33,425(30.6)	14,201(45.3)	11,149(25.8)
45–54	253,691(17.4)	37,383(11.4)	196,720(20.8)	6,934(6.4)	7,695(24.5)	4,959(11.5)
55–64	178,689(12.3)	13,479(4.1)	161,056(17.0)	890(0.8)	1,949(6.2)	1,315(3.0)
≥ 65	167,111(11.5)	5,631(1.7)	160,504(17.0)	222(0.2)	310(1.0)	444(1.0)
Marital status*						
Unmarried	463,151(32.0)	213,014(65.4)	186,750(19.8)	41,494(40.0)	1,714(5.5)	20,179(47.1)
Married	626,555(43.3)	65,580(20.1)	481,185(51.0)	42,408(40.9)	22,263(71.4)	15,119(35.3)
Divorced or widowed	356,587(24.7)	47,270(14.5)	274,700(29.1)	19,830(19.1)	7,219(23.1)	7,568(17.7)
Ethnicity						
Han	1,174,538(80.6)	307,500(94.1)	745,794(78.8)	55,968(51.3)	30,757(98.1)	34,519(80.0)
Minority	282,680(19.4)	19,187(5.9)	201,060(21.2)	53,211(48.7)	601(1.9)	8,621(20.0)
Education*						
Primary school or illiteracy	536,077(36.9)	18,715(5.7)	431,623(45.7)	55,234(51.7)	19,338(62.4)	11,167(26.1)
Middle school	485,193(33.4)	83,626(25.6)	331,696(35.1)	43,424(40.7)	10,843(35.0)	15,604(36.5)
College and above	431,023(29.7)	224,301(68.7)	181,799(19.2)	8,125(7.6)	821(2.6)	15,977(37.4)
Diagnosis locations						
Hospitals	778,841(53.4)	124,865(38.2)	613,269(64.8)	20,581(18.9)	3,225(10.3)	16,901(39.2)
VCT	375,190(25.7)	142,904(43.7)	188,389(19.9)	22,086(20.2)	10,459(33.4)	11,352(26.3)
Others	303,187(20.8)	58,918(18.0)	145,196(15.3)	66,512(60.9)	17,674(56.4)	14,887(34.5)
Regional distribution						
East	333,861(22.9)	160,020(49.0)	144,622(15.3)	14,222(13.0)	768(2.4)	14,229(33.0)
Central	286,762(19.7)	84,646(25.9)	159,188(16.8)	4,542(4.2)	30,015(95.7)	8,371(19.4)
West	836,595(57.4)	82,021(25.1)	643,044(67.9)	90,415(82.8)	575(1.8)	20,540(47.6)
Years of diagnosis						
1989–2012	349,629(24.0)	38,057(11.6)	179,199(18.9)	81,904(75.0)	31,218(99.6)	19,251(44.6)
2013–2016	377,871(25.9)	103,673(31.7)	247,105(26.1)	17,257(15.8)	99(0.3)	9,737(22.6)
2017–2019	390,562(26.8)	97,058(29.7)	276,983(29.3)	8,333(7.6)	17(0.1)	8,171(18.9)
2020–2022	339,156(23.3)	87,899(26.9)	243,567(25.7)	1,685(1.5)	24(0.1)	5,981(13.9)
Migration						
No	1,147,775(78.8)	183,442(56.2)	806,292(85.2)	96,574(88.5)	30,529(97.4)	30,938(71.7)
Yes	309,443(21.2)	143,245(43.8)	140,562(14.8)	12,605(11.5)	829(2.6)	12,202(28.3)
Residence*						
Urban	873,814(62.0)	266,983(82.6)	521,190(57.2)	49,240(48.4)	9,030(29.4)	27,371(64.7)
Rural	535,000(38.0)	56,046(17.4)	389,930(42.8)	52,445(51.6)	21,674(70.6)	14,905(35.3)

Asterisk indicates missing values

*Abbreviations*: *IDU* injection drug users, *FPD* former plasma donors, *MSM* men who have sex with men, *HRHB* high-risk heterosexual behavior

### Comparison of the occurrence of HRHB among different populations

Among all 43,140 individuals reporting multiple contact histories, 42,258 (98.0%) engaged in HRHB, with only 5,545 (13.1%) of those presumed to have become infected through heterosexual behavior. The transmission routes of the other individuals are documented in Table 2 (transmission routes for 340 individuals remain unknown). Prior to 2012, the majority of HIV-infected individuals engaging in HRHB were IDUs. However, subsequent to this year, the majority shifted to MSM. HIV-infected FPDs who had engaged in HRHB were only reported before or after 2004. Among all HIV-infected IDUs, MSM, and FPDs, their respective proportions of engagement in HRHB were 11.8%, 5.7% and 6.2% (χ2 = 5057.0, *P* < 0.001).

**Table 2:** Numbers and proportions of HRHB among HIV-infected IDUs, MSM, and FPDs in China

Years	Numbers			Proportions(%)		
	**IDUs**	**MSM**	**FPDs**	**IDUs**	**MSM**	**FPDs**
**1989–1999**	74	2	22	1.9	28.6	3.0
**2000**	27	0	5	2.6	0.0	3.9
**2001**	47	0	3	3.6	0.0	0.9
**2002**	66	0	7	5.1	0.0	1.4
**2003**	87	4	158	5.2	16.7	7.9
**2004**	323	4	1,360	5.6	7.0	8.0
**2005**	1,644	28	218	16.5	13.8	6.2
**2006**	1,414	50	126	15.1	10.4	5.9
**2007**	1,479	131	88	14.0	11.9	4.5
**2008**	1,743	234	25	15.6	8.5	1.5
**2009**	1,373	368	18	12.4	7.9	1.4
**2010**	1,100	547	11	12.5	8.0	1.2
**2011**	966	798	15	11.7	7.8	1.9
**2012**	655	1,003	8	9.8	6.7	1.7
**2013**	542	1,134	2	9.5	6.0	4.9
**2014**	508	1,539	2	10.0	5.9	10.5
**2015**	397	1,730	0	9.2	5.4	0.0
**2016**	371	1,776	0	9.3	5.3	0.0
**2017**	445	1,811	0	11.6	5.4	-
**2018**	462	1,966	0	11.4	5.8	-
**2019**	270	1,982	0	16.2	5.7	-
**2020**	193	1,851	0	20.0	6.0	-
**2021**	137	1,743	0	19.2	5.1	-
**2022**	89	1,192	0	20.3	4.3	-
**total**	**14,412**	**19,893**	**2,068**	**11.8**	**5.7**	**6.2**

*Abbreviations*: *IDU* injection drug users, *FPD* former plasma donors, *MSM* men who have sex with men

### Factors associated with HRHB among MSM

Because most HIV-infected individuals who reported multiple contact histories in recent years were mainly MSM, we analyzed risk factors affecting the occurrence of HRHB in MSM. Among all the MSM who were assessed getting infected by homosexual sex, multivariate logistic regression models highlighted age (25–34 years: AOR = 1.29, 35–44 years: AOR = 1.22), marital status (married: AOR = 1.23, divorced or widowed: AOR = 1.17), ethnic minority status (AOR = 1.29), hospital diagnoses (AOR = 1.91), lower education levels (primary or illiteracy: AOR = 1.32, junior high school: *AOR* = 1.22), and rural residence (*AOR* = 1.12) as significant predictors of HRHB. However, the risk of HRHB decreased when age ≥ 55 years (55–64 years: AOR = 0.82, ≥ 65 years: AOR = 0.64). The P-values of the above variables are all less than 0.05. Our findings suggest that the risk of HRHB has been decreasing over time (χ2 trend = 215.4, *P* < 0.001). Specifically, the risk among HIV-infected MSM identified after 2020 was 0.6 times (0.58 to 0.64) lower than among those identified prior to 2012 (Table 3).

**Table 3:** Factors associated with HRHB among HIV-infected MSM in China

Factors	Total	HRHB(%)	univariate analysis		multivariate analysis	
			*OR*(95%*CI*)	*P*	*AOR*(95%*CI*)	*P*
Total	347,041	19,893(5.7)				
Age(years)						
15–24	98,716	4,475(4.5)	1		1	
25–34	126,341	7,751(6.1)	1.38(1.33–1.43)	< 0.001	1.29(1.24–1.35)	< 0.001
35–44	61,968	4,190(6.8)	1.53(1.46–1.60)	< 0.001	1.22 (1.16–1.29)	< 0.001
45–54	39,892	2,459(6.2)	1.38(1.32–1.46)	< 0.001	1.02(0.95–1.08)	0.614
55–64	14,238	756(5.3)	1.18(1.10–1.28)	< 0.001	0.82(0.75–0.90)	< 0.001
≥ 65	5,886	262(4.5)	0.98(0.86–1.11)	0.769	0.64(0.55–0.73)	< 0.001
Marital status*						
Unmarried	225,100	11,760(5.2)	1		1	
Married	70,553	4,888(6.9)	1.35(1.31–1.40)	< 0.001	1.23(1.18–1.29)	< 0.001
Divorced or widowed	50,541	3,206(6.3)	1.23(1.18–1.28)	< 0.001	1.17(1.11–1.23)	< 0.001
Ethnicity						
Han	326,395	18,426(5.6)	1		1	
Minority	20,646	1,467(7.1)	1.28(1.21–1.35)	< 0.001	1.29(1.22–1.37)	< 0.001
Education*						
Primary school or illiteracy	20,191	1,456(7.2)	1.42(1.34–1.50)	< 0.001	1.32(1.24–1.40)	< 0.001
Middle school	89,864	6,134(6.8)	1.34(1.30–1.38)	< 0.001	1.22(1.18–1.27)	< 0.001
College and above	236,968	12,302(5.2)	1		1	
Diagnosis locations						
VCT	149,141	6,139(4.1)	1		1	
Hospitals	135,399	10,383(7.7)	1.94(1.87–2.00)	< 0.001	1.91(1.85–1.98)	< 0.001
Others	62,501	3,371(5.4)	1.33(1.27–1.39)	< 0.001	1.25(1.19–1.30)	< 0.001
Regional distribution						
East	170,671	10,363(6.1)	1.22(1.18–1.27)	< 0.001	1.17(1.13–1.22)	< 0.001
Central	87,132	5,048(5.8)	1.16(1.12–1.21)	< 0.001	1.11(1.06–1.16)	< 0.001
West	89,238	4,482(5.0)	1		1	
Years of diagnosis						
1998–2012	41,428	3,169(7.6)	1		1	
2013–2016	110,019	6,179(5.6)	0.72(0.69–0.75)	< 0.001	0.70(0.67–0.73)	< 0.001
2017–2019	102,907	5,759(5.6)	0.72(0.68–0.75)	< 0.001	0.69(0.66–0.72)	< 0.001
2020–2022	92,687	4,786(5.2)	0.66(0.63–0.69)	< 0.001	0.61(0.58–0.64)	< 0.001
Migration*						
No	195,116	11,393(5.8)	1		1	
Yes	151,925	8,500(5.6)	0.96(0.93–0.98)	0.002	1.03 (1.00–1.07)	0.061
Residence*						
Urban	283,292	15,904(5.6)	1		1	
Rural	59,891	3,793(6.3)	1.14(1.10–1.18)	< 0.001	1.12(1.08–1.17)	< 0.001

Asterisk indicates missing values

*Abbreviations*: *OR* odds ratio, *CI* confidence interval, *AOR* adjusted odds, *HRHB* high-risk heterosexual behavior, *MSM* men who have sex with men, *VCT* voluntary counseling and testing

## Discussion

This study found that the proportion of multiple contact histories among individuals having acquired HIV through the four dominant transmission routes in China was 3% since 1989. Before 2012, most of them were IDUs who have ever engaged in HRHB, followed by FPDs; after 2012, most were MSM who have ever engaged in HRHB. Despite the fact that the vast majority (98%) of them ever engaged in HRHB, only a small proportion (13%) were judged to have been infected through heterosexual behavior after behavioral risk judgment by the investigators, which validates the hypothesis of an early spread of HIV from the IDUs and FPDs to the general population [9].

Regarding transmission routes, IDUs have reported the highest incidence of HRHB. Particularly, a large number of HIV-infected IDUs was diagnosed from 2005 to 2011, with approximately 12% of these individuals engaging in HRHB. This timeframe also saw the most rapid increase in reported heterosexual infections [18]. Sentinel surveillance data from this period revealed an HIV-positive rate of about 3% among drug users, and a syphilis antibody-positive rate ranging between 4 and 5% [19]. This data reinforces the increased risk associated with IDU populations transmitting HIV to others through sexual activity. Despite recent effective control of drug injection transmission, a high proportion of the IDU population is diagnosed late [20], combined with the rate of HRHB being as high as 20% recent years, the risk of HIV spread still exists.

Although the HIV-infected FPDs were only reported in a short period, their impact on the AIDS epidemic in China was believed to be significant. The outbreak of HIV infection among FPDs was occurred in 1995, and the screening for HIV among all the FPDs was in late 2004, by which time many of those infected had died [21, 22]. For tens of thousands FPDs, the duration from infection to diagnosis was nearly 10 years, in which time they were capable of passing on their infection due to virally unsuppressed without ART [4]. The finding in our study that 6.2% of FPDs ever engaged HRHB partially explained that the epidemic in central China quickly acquired the characteristics of a heterosexual epidemic [23].

As control over drug injection transmission and the elimination of plasma transmission progress, their impact on the overall epidemic within the general population is steadily decreasing, if not virtually eradicated. However, the bridging role played by MSM remains steadily persistent. This study discovered that 35% of HIV-infected MSM are currently or have formerly been in a marital relationship, and approximately 5.7% have engaged in HRHB.

Previous studies revealed that prior to diagnosis, 7.6% of infected MSM had transmitted the disease to their spouse [24], and less than 40% disclosed their HIV status post-diagnosis [25]. This suggests a higher probability of HIV-positive MSM transmitting the disease to their female partners both pre and post diagnosis. What’s more, beside a high marriage rate in MSM, a high rate of HRHB in MSM was much more concerning. Not just because the bi-sexual individuals engage in unprotected sex at higher rates than homosexuals [26, 27], but also the rate of notification to non-regular partners is significantly lower than to spouses or regular partners [28]. Thus, there is an increased risk of MSM transmitting HIV to the general population through non-marital heterosexual behavior.

Consistent with prior research, the MSM aged 25–44 years have a high risk of HRHB comparing who < 25 years [29]. However, we initially found out that the odds reversed when they aged above 55 years old. This situation may relate to the decreased need for sex in older men [30]. What’s more, this work establishes for the first time that individuals identified in hospitals demonstrate the highest risk for engaging in HRHB. This may stem from passive testing-detected individuals displaying inadequate HIV testing awareness and deficient HIV knowledge. In contrast, those identified through VCT exhibit more risk awareness and will voluntarily mitigate risky sexual behavior following the consultation [31, 32]. Despite the gradually decreasing HRHB risk among MSM, the high potential for transmission persists, given the extensive population of bisexual individuals who function as a "bridge".

This study carries two potential constraints. Initially, the misclassification of the transmission route may occur due to reliance on self-reported contact history from the infected individual. Second, contact history is sensitive information and there may be intentional concealment or recollection bias by the HIV-infected individual. Consequently, the proportion of multiple contact histories could potentially be underestimated.

## Conclusions

The risk of HIV transmission among distinct populations is heightened. Our research indicates a pronounced prevalence of high-risk heterosexual behaviors among these groups, particularly among MSM who have witnessed substantial infections in recent years. A targeted response involving harm-reduction strategies and health education for bisexual MSM and their female partners is critical to curb further transmission of HIV into the larger population.

## Data Availability

The datasets used and analyzed during the current study are available from the corresponding author on reasonable request.

## Abbreviations

HIV: Human immunodeficiency virus
AIDS: Acquired immune deficiency syndrome
MSM: Men who have sex with men
IDU: Injection drug users
FPD: Former plasma donors
HRHB: High-risk heterosexual behavior
OR: Odds ratio
CI: Confidence interval
AOR: Adjusted odds
VCT: Voluntary counseling and testing

## References

[1] Han MJ (2023). Analysis of HIV epidemic and prospects for prevention and control in China. Chin J AIDS STD.

[2] He N (2021). Research Progress in the Epidemiology of HIV/AIDS in China. China CDC Wkly.

[3] Rodger AJ, Cambiano V, Bruun T (2019). Risk of HIV transmission through condomless sex in serodifferent gay couples with the HIV-positive partner taking suppressive antiretroviral therapy (PARTNER): final results of a multicentre, prospective, observational study. Lancet.

[4] Skarbinski J, Rosenberg E, Paz-Bailey G (2015). Human immunodeficiency virus transmission at each step of the care continuum in the United States. JAMA Intern Med.

[5] Li AH, Wu ZY, Jiang Z, McGoogan JM, Zhao Y, Duan S (2018). Duration of Human Immunodef iciency Virus Infection at Diagnosis among New Human Immunodef iciency Virus Cases in Dehong, Yunnan, China, 2008–2015. Chin Med J (Engl).

[6] Wu Z, McGoogan JM, Detels R (2021). The Enigma of the Human Immunodeficiency Virus (HIV) Epidemic in China. Clin Infect Dis.

[7] Lu L, Jia M, Ma Y (2008). The changing face of HIV in China. Nature.

[8] Ding Y, Ma Z, He J (2019). Evolving HIV Epidemiology in Mainland China: 2009–2018. Curr HIV/AIDS Rep.

[9] He X, Xing H, Ruan YH, Hong KX, Cheng CL, Hu YY (2012). A comprehensive mapping of HIV-1 genotypes in various risk groups and regions across China based on a nationwide molecular epidemiologic survey[J/OL]. PLoS ONE.

[10] Tang HL, Lv F (2007). Role of bridge population in the transmission of human immunodeficiency virus. Chin J Epidemiol.

[11] Mao Y, Wu Z, Poundstone K, Wang C, Qin Q, Ma Y (2010). Development of a unified web-based national HIV/AIDS information system in China. Int J Epidemiol.

[12] Xing J, Li YG, Tang W (2014). HIV/AIDS epidemic among older adults in China during 2005–2012: results from trend and spatial analysis. Clin Infect Dis.

[13] Qin Q, Guo W, Tang W (2017). Spatial Analysis of the Human Immunodeficiency Virus Epidemic among Men Who Have Sex with Men in China, 2006–2015. Clin Infect Dis.

[14] Patel P, Borkowf CB, Brooks JT, Lasry A, Lansky A, Mermin J (2014). Estimating per-act HIV transmission risk: a systematic review. AIDS.

[15] Dong Z, Ma L, Cai C, Gao GF, Lyu F (2021). Demographic features of identified PLWHA infected through commercial and nonmarital noncommercial heterosexual contact in China from 2015 to 2018: a retrospective cross-sectional study. BMC Infect Dis.

[16] Li DM, Wang L, Gao S, Wang Z, Cui ZL, Song LP (2010). Study on the natural history of HIV among former commercial plasma donors caused by contaminated plasma donation in central China. Chin J Epidemiol.

[17] Bulletin of main data of the first national Economic census (No. 1) [EB/OL]. [2023–06–28]. http://www.stats.gov.cn/zt_18555/zdtjgz/zgjjpc/dycjjpc/cgfb/202303/t20230306_1935043.htm.

[18] Wu ZY (2018). New characteristics of sexual transmission of AIDS in China and challenges in prevention and treatment. Chin J Epidemiol.

[19] Ge L, Li DM, Li PL, Guo W, Cui Y (2017). Population specific sentinel surveillance for HIV infection, syphilis and HCV infection in China, during 2010–2015. Dis Surv.

[20] Tang H, Cai C, Cui Y, Qin QQ, Lyu F (2020). Epidemiological Characteristics of Newly Diagnosed Cases of HIV through Injection Drug Use — China, 2012–2019[J/OL]. China CDC Weekly.

[21] Dou Z, Chen RY, Wang Z (2010). HIV-infected former plasma donors in rural Central China: from infection to survival outcomes, 1985–2008. PLoS ONE.

[22] Sun X, Wang N, Li D (2007). The development of HIV/AIDS surveillance in China. AIDS.

[23] Liang Y, Li N, Sun DY, Fan PY, Yang WJ, Nie YG (2020). Characteristics of newly reported HIV/AIDS cases in Henan province, 2010–2018. Chin J Epidemiol.

[24] Li J, Han J, Xu J, Tang HL, Mao YR (2017). Status of marriage and HIV transmission between couples in newly reported HIV cases before diagnosis was made, among men who have sex with men in China, 2014. Chin J Epidemiol.

[25] Chi YY, Huang DP, Lindgren T, Goldsamt L, Zhou J, Ren Y (2022). The association between HIV disclosure, spousal testing and unprotected vaginal intercourse within marriage among HIV positive married MSM in China[J/OL]. AIDS Care.

[26] Feinstein BA, Moran KO, Newcomb ME, Newcomb ME, Mustanski B (2019). Differences in HIV Risk Behaviors Between Self-Identified Gay and Bisexual Young Men Who are HIV-Negative[J/OL]. Arch Sex Behav.

[27] Shen H, Tang S, Mahapatra T (2016). Condomless Vaginal Intercourse and Its Associates among Men Who Have Sex with Men in China[J/OL]. PLoS ONE.

[28] Peng WW, Song XH, Zhang C, Chen YQ, Zhou QD, Välimäki MA (2022). The proportion of HIV disclosure to sexual partners among people diagnosed with HIV in China: A systematic review and meta-analysis[J/OL]. Front Public Health.

[29] Liao M, Wang M, Shen X (2015). Bisexual Behaviors, HIV Knowledge, and Stigmatizing/Discriminatory Attitudes among Men Who Have Sex with Men. PLoS ONE.

[30] Zhou C, Cai C, Hu M, Wang XT, Li JX, Zhou HY (2021). Sexual Needs and Sexual Behaviors among Married Male Residents Aged 50 and Older in Rural Areas of Two Provinces. China Chin J AIDS STD.

[31] Chen J, Xu J, Zhou Y (2022). HIV Detection and Delayed Diagnosis: A Time Series Analysis in China[J/OL]. Int J Environ Res Public Health.

[32] Costa AB, Viscardi LH, Feijo M, Fontanari AM (2022). HIV Voluntary Counseling and Testing (VCT-HIV) effectiveness for sexual risk-reduction among key populations: A systematic review and meta-analysis[J/OL]. EClinicalMedicine.

