# Correction: Behavioral verification and risk factors of HIV cross-population transmission in China: analysis of national surveillance data 1989–2022

**DOI:** 10.1186/s12879-024-09045-1

**Published:** 2024-02-21

**Authors:** Chang Cai, Houlin Tang, Qianqian Qin, Yichen Jin, Fan Lyu

**Affiliations:** 1 Division of Epidemiology, National Center for AIDS/STD Control and Prevention, Chinese Center for Disease Control and Prevention, Beijing, 102206 China; 2 National Key Laboratory of Intelligent Tracking and Forecasting for Infectious Diseases, National Center for AIDS/STD Control and Prevention, Chinese Center for Disease Control and Prevention, Beijing, 102206 China

**Correction: BMC Infectious Diseases 24, 49 (2024)**

**https://doi.org/10.1186/s12879-023-08956-9**

Following publication of the original article [1], we have been notified that in Table 1, under the variable "Diagnosis locations", the VCT and Hospitals need to change positions.

The original article has been corrected.
